# Sex Difference of Ribosome in Stroke-Induced Peripheral Immunosuppression by Integrated Bioinformatics Analysis

**DOI:** 10.1155/2020/3650935

**Published:** 2020-12-03

**Authors:** Jian-Qin Xie, Ya-Peng Lu, Hong-Li Sun, Li-Na Gao, Pei-Pei Song, Zhi-Jun Feng, Chong-Ge You

**Affiliations:** ^1^Department of Anesthesiology, Lanzhou University Second Hospital, Lanzhou, Gansu 730030, China; ^2^Laboratory Medicine Center, Lanzhou University Second Hospital, Lanzhou, Gansu 730030, China; ^3^The Second Clinical Medical College of Lanzhou University, Lanzhou, Gansu 730030, China

## Abstract

Ischemic stroke (IS) greatly threatens human health resulting in high mortality and substantial loss of function. Recent studies have shown that the outcome of IS has sex specific, but its mechanism is still unclear. This study is aimed at identifying the sexually dimorphic to peripheral immune response in IS progression, predicting potential prognostic biomarkers that can lead to sex-specific outcome, and revealing potential treatment targets. Gene expression dataset GSE37587, including 68 peripheral whole blood samples which were collected within 24 hours from known onset of symptom and again at 24-48 hours after onset (20 women and 14 men), was downloaded from the Gene Expression Omnibus (GEO) datasets. First, using Bioconductor R package, two kinds of differentially expressed genes (DEGs) (nonsex-specific- and sex-specific-DEGs) were screened by follow-up (24-48 hours) vs. baseline (24 hours). 30 nonsex-specific DEGs (1 upregulated and 29 downregulated), 79 female-specific DEGs (25 upregulated and 54 downregulated), and none of male-specific DEGs were obtained finally. Second, bioinformatics analysis of female-specific DEGs was performed. Gene Ontology (GO) functional annotation analysis shows that DEGs were mainly enriched in translational initiation, cytosolic ribosome, and structural constituent of ribosome. Kyoto Encyclopedia of Genes and Genomes (KEGG) pathway enrichment analysis shows that the top 6 enrichment pathways are ribosome, nuclear factor-­kappa B (NF-kappa B) signaling pathway, apoptosis, mineral absorption, nonalcoholic fatty liver disease, and pertussis. Three functional modules were clustered in the protein–protein interaction (PPI) network of DEGs. The top 10 key genes of the PPI network constructed were selected, including *RPS14*, *RPS15A*, *RPS24*, *FAU*, *RPL27*, *RPL31*, *RPL34*, *RPL35A*, *RSL24D1*, and *EEF1B2*. Sex difference of ribosome in stroke-induced peripheral immunosuppression may be the potential mechanism of sex disparities in outcome after IS, and women are more likely to have stroke-induced immunosuppression. RPS14, RPS15A, RPS24, FAU, RPL27, RPL31, RPL34, RPL35A, RSL24D1, and EEF1B2 may be novel prognostic biomarkers and potential therapeutic targets for IS.

## 1. Introduction

Stroke was the second largest cause of death worldwide after ischemic heart disease and the second most common cause of worldwide disability-adjusted life years (DALYs). There were 80.1 million prevalent cases of stroke, which 84.4% contributed ischemic stroke (IS) and 13.7 million new stroke cases globally in 2016 [1]. The mean global lifetime risk of stroke increased from 22.8% in 1990 to 24.9% in 2016 [2]. On average, stroke occurs every 40 seconds, and stroke caused death every 3 minutes and 42 seconds in the United States [3]. Stroke is characterized by significant sex differences. The difference of sex on various aspects of stroke, such as risk factors, epidemiology, incidence, pathogenesis, mortality, prognosis, clinical presentation, and response to treatment, has been extensively investigated in the past years. Although a large number of experiments in vivo have proved that estrogen has a protective effect on the brain [4, 5], there is sufficient clinic evidence that women more often had poor functional outcome compared with men, and this difference was not dependent on age [6, 7]. For example, a study found that young female rodents have smaller infarct volume and better cerebral blood flow (CBF) than age-matched males, and these sex differences reverse with aging [8]. Meanwhile, in a large European multicenter prospective observational clinical cohort study, researchers found that female sex remained associated with poor functional 3-month outcome after adjustments for baseline differences through analyzing 9495 acute IS patients who were treated with intravenous thrombolysis (IVT), and this finding was not dependent on age and could not be explained by a higher bleeding risk or mortality rate in women [7]. This study is basically consistent with the results of a previous study. The previous study based 47209 patients (>18 years) with ischemic stroke or clinically defined transient ischemic attack found that women had a worse functional outcome at 3-month follow-up, but a lower mortality after correcting for confounders [6]. So, not only sex hormones but also sex chromosomes may contribute to the different outcome following stroke. Therefore, it is necessary to further study the sex specificity of stroke, so as to provide basis for accurate evaluation of prognosis and formulation of treatment strategies.

In recent years, more and more attention has been paid to the pathogenesis of stroke-induced immunodeficiency syndrome (SIDS) [9] and stroke-associated infection (SAI). The inflammatory response after stroke is an important way to remove the necrotic tissue of brain injury, but excessive inflammatory response can also cause secondary inflammatory damage. Although stroke-induced immunosuppression is a necessary protective feedback mechanism for the body to resist inflammatory injury, it is also an important risk factor for stroke patients, which could reduce their immune defense, increase the susceptibility to infection, lead to serious infectious complications, and increase stroke mortality. Therefore, the balance between proinflammation and anti-inflammation is the crucial to the recovery of IS patients. However, little is known about whether stroke-induced immunodepression has difference between female and male.

Because of the dynamic nature of transcriptional regulation, RNA levels represent not only features encoded in the genome but also the influence of the environment [10]. Thus, some investigators used gene expression profiling as the starting point for biomarker discovery and identification of disease mechanisms [11]. Microarrays based on high-throughput platforms for the profiling of genome-wide expression emerge as a promising and efficient tool for inferring biological relevancy. It is especially suitable for the study of the dynamic development process of complex diseases such as IS [12]. However, few studies have been done on the sexually dimorphic to peripheral immune response in IS progression at RNA level. This study tried to screen out the sex-specific differentially expressed genes (DEGs) in the peripheral immune response in IS progression. Normally in sexually dimorphic study, sex-specific DEGs (male specific and female specific) are main research target. But to further explore the potential connection between sex-specific DEGs and nonsex-specific DEGs, two kinds of DEGs, nonsex-specific DEGs and sex-specific DEGs (male specific and female specific), were screened in this study.

In this study, we have downloaded one original microarray dataset GSE37587 from the Gene Expression Omnibus (GEO) datasets, including 68 peripheral whole blood samples which were collected within 24 hours from known onset of symptom and again at 24-48 hours after onset (20 women and 14 men). Using Bioconductor R package, nonsex-specific and sex-specific DEGs were got by follow-up (24-48 hours) vs. baseline (24 hours) with 30 nonsex-specific DEGs, 79 female-specific DEGs, and 0 male-specific DEGs. Then, Gene Ontology (GO) functional annotation and Kyoto Encyclopedia of Genes and Genomes (KEGG) pathway enrichment on female-specific DEGs were performed by the R software. Finally, the STRING online database protein–protein interaction (PPI) network was used to analyze the association of female-specific DEGs and discover the molecular interactions involved in IS progression. Meanwhile, according to the PPI network result, molecular complex detection (MCODE) modules was established and key gene analysis was performed by Cytoscape for identifying key genes of optimal significance. In conclusion, sex-specific DEGs associated with the IS progression were screened and an integrated analysis was conducted. Our study is aimed at identifying the gender dimorphic to peripheral immune response in IS progression, predicting potential prognostic biomarkers that can lead to sex-specific prognosis, and revealing potential treatment targets.

## 2. Materials and Methods

### 2.1. Microarray Data

The GSE37587 gene expression profile series matrix file(s) and SOFT formatted family file(s) were downloaded from GEO datasets (https://www.ncbi.nlm.nih.gov/geo/), which platform is the GPL6883 Illumina HumanRef-8 v3.0 expression beadchip. Robust Multichip Average (RMA) normalization collation had been done with the series matrix file(s), including background correction, quantile normalization, and summarization. A total of 68 peripheral whole blood samples were included in the dataset, which were collected from *n* = 34, and magnetic resonance imaging (MRI) diagnosed IS patients ≥ 18 years of age within 24 hours from known onset of symptom and again at 24-48 hours after onset. IS patients, whose ethnicity are Caucasian, included 20 women with mean age of 72.15 ± 15.951 years and 14 men with mean age of 71.64 ± 13.054 years.

### 2.2. Screening for DEGs

The analysis was carried out with the R language software (version 3.6.2). First, the dataset was normalized by log_2_ transformation. Second, the 24,526 probe IDs were converted into gene symbols according to the SOFT formatted family file(s). If multiple probes correspond to the same gene, only the probe with the highest mean expression value is retained, and 18631 genes were finally obtained. Third, the DEG analysis was performed using the Linear Models for Microarray Data (limma) package (http://www.bioconductor.org/). DEGs were selected with thresholds of ∣log_2_ (fold change) (FC) | >0.6 and adjusted *p* value <0.05. Two kinds of DEGs were screened: (1) nonsex-specific DEGs by follow-up (24-48 hours) vs. baseline (24 hours) and (2) sex-specific DEGs by female follow-up (24-48 hours) vs. female baseline (24 hours) and male follow-up (24-48 hours) vs. male baseline (24 hours).

### 2.3. Bioinformatics Analysis

#### 2.3.1. GO Enrichment Analysis of Female-Specific DEGs

For the sets of female-specific DEGs, GO analysis was performed using clusterProfiler in R package with *p*AdjustMethod = ^“^BH,^”^, *p*valueCutoff = 0.05, and *q*valueCutoff = 0.2 [13]. Then, we used the function simplify to remove the redundancy of enriched GO terms.

#### 2.3.2. KEGG Pathway Enrichment Analysis of Female-Specific DEGs

KEGG pathway enrichment analysis was performed using clusterProfiler with *p*AdjustMethod = ^“^BH,^”^, and *p* value <0.05 was considered statistically significant.

#### 2.3.3. Integration of the PPI Network Analysis

To evaluate the interactive relationships among the female-specific DEGs, we mapped them to the STRING database (http://string-db.org/) (version 11.0), and only the interaction score > 0.4 (medium confidence) was considered significant. Interactions in STRING are derived from five main sources: genomic context predictions, high-throughput lab experiments, (conserved) coexpression, automated text mining, and previous knowledge in databases. The PPI network was visualized by the Cytoscape software (version 3.7.2).

#### 2.3.4. Functional Module Identification in the PPI Network

MCODE, molecular complex detection, a Cytoscape plug-in, clusters a given network based on topology to find densely connected. MCODE was used to identify functional module in the PPI network constructed with the default settings of degree cutoff at 2, node density cutoff at 0.1, node score cutoff at 0.2, k-core at 2, and maximum depth at 100.

#### 2.3.5. Selection of Key Genes

CytoHubba, a Cytoscape plug-in, was used to get the top 10 key genes in the PPI network constructed with the highest MCC score.

## 3. Results and Discussion

### 3.1. Results

#### 3.1.1. Identification of Two Kinds of DEGs

**Figure 1:**
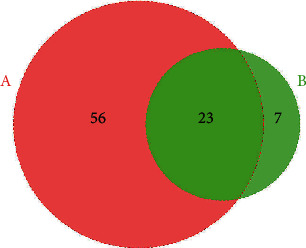
The coexpression distribution of female-specific and nonsex-specific DEGs. Red A represents female-specific DEGs. Green B represents nonsex-specific DEGs. DEGs: differentially expressed genes.

**Figure 2:**
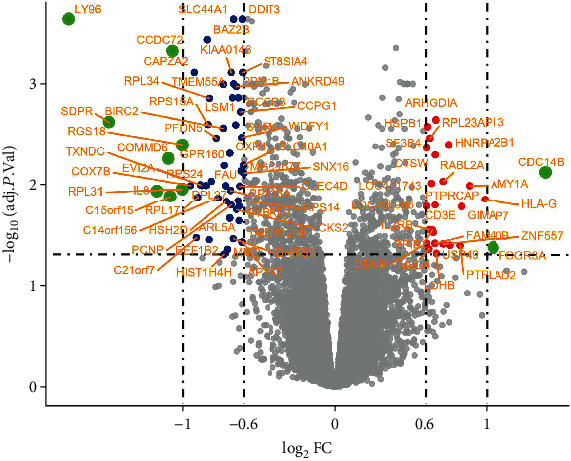
Female-specific differential expression of data between follow-up and baseline. The red points represent upregulated DEGs with log_2_ FC > 0.6 and adjusted *p* value <0.05. The blue points represent downregulated DEGs with log_2_ FC < −0.6 and adjusted *p* value <0.05. The green points represent DEGs with ∣log_2_ FC | >1 and adjusted *p* value <0.05. The grey points represent genes with ∣log_2_ FC | <0.6. The gene symbols of the DEGs are marked in orange. *C15orf15* is also known as *RSL24D1*. DEGs: differentially expressed genes; FC: fold change.

**Figure 3:**
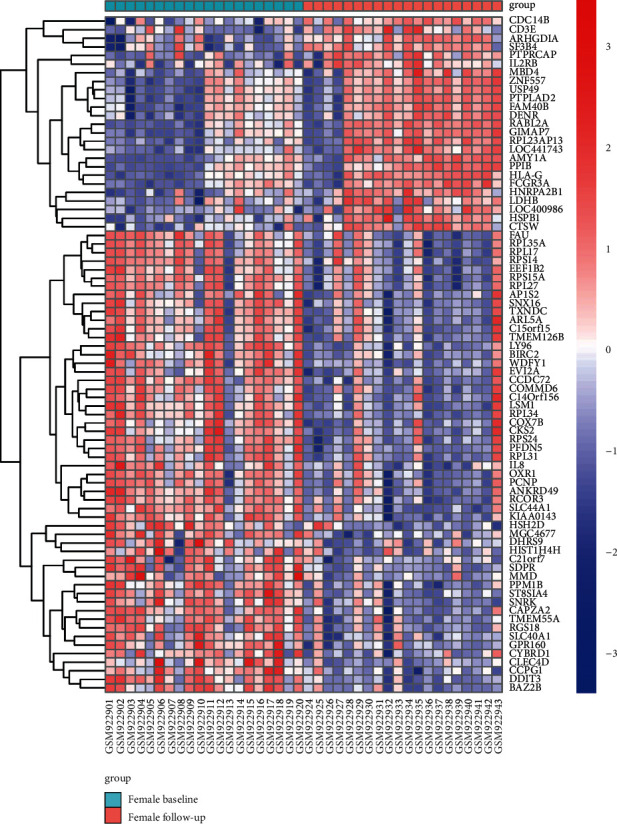
Cluster heatmap of the female-specific DEGs. Red indicates upregulation of gene expression. Blue indicates downregulation of gene expression. White indicates no significant change in gene expression. *C15orf15* is also known as *RSL24D1*.

The GSE37587 dataset contained 68 peripheral whole blood samples, which were collected from 20 women and 14 men within 24 hours (baseline) from known onset of IS symptom and again at 24-48 hours (follow-up) after onset. 30 nonsex-specific DEGs (1 upregulated and 29 downregulated) were obtained by comparison of 34 follow-up and 34 baseline samples. 79 female-specific DEGs (25 upregulated and 54 downregulated) were obtained by comparison of 20 female follow-up and 20 female baseline samples. And none of male-specific DEGs were obtained by comparison of 14 male follow-up and 14 male baseline samples. The details of all DEGs are shown in Table 1. The unique one upregulated gene in nonsex-specific DEGs is also upregulated in female-specific DEGs. The coexpression distribution of female-specific and nonsex-specific DEGs is shown in Venn diagram (Figure 1). Seven genes unique to nonsex-specific DEGs include *AKAP7*, *RPL39*, *RPL26*, *MBNL2*, *HES4*, *MASK*, and *HINT1*. The female-specific DEGs are shown in the volcano plot (Figure 2), and the cluster heatmap is shown in Figure 3, which is, respectively, drawn by ggplot2 package and heatmap function in the R software.

#### 3.1.2. Bioinformatics Analysis of Female-Specific DEGs

#### 3.1.3. GO Enrichment Analysis of Female-Specific DEGs

**Figure 4:**
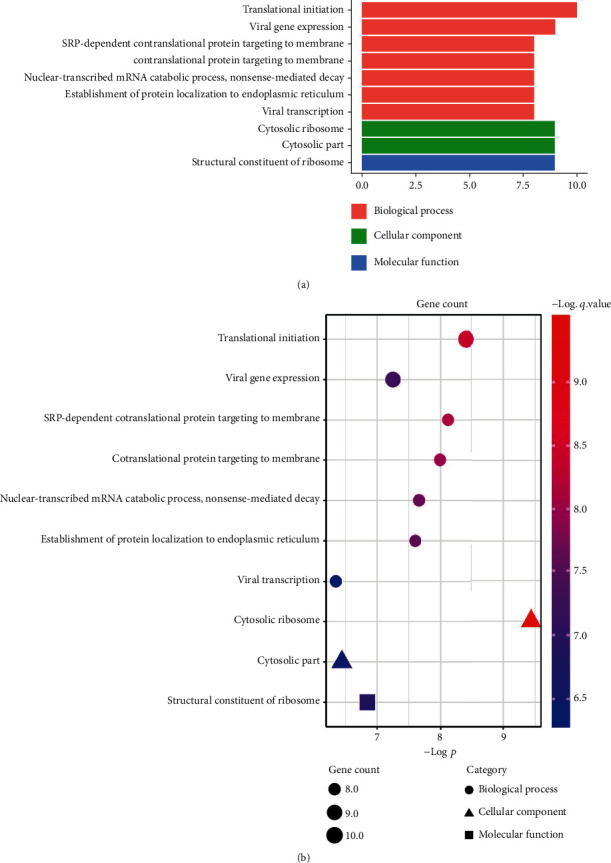
GO enrichment analysis of female-specific DEGs. (a) Gene count in biological process, cell component, and molecular function. (b) GO enrichment significance terms of female-specific DEGs in different functional groups.

**Figure 5:**
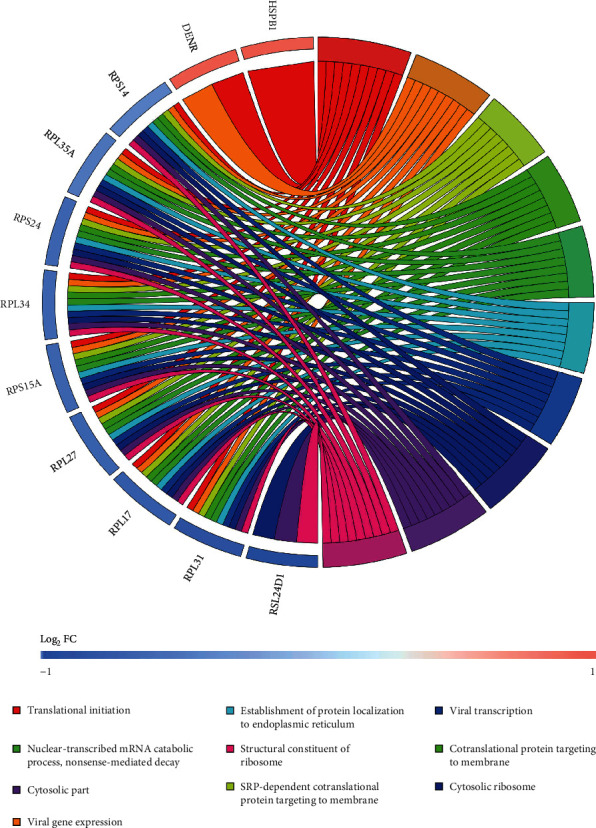
Distribution of female-specific DEGs in different GO enrichment functions.

GO function annotation analysis was performed using clusterProfiler in R package, which was divided into three parts: biological process (BP), cell component (CC), and molecular function (MF). Those results were considered statistically significant if *p* value <0.05 and *q* value <0.2. The redundancy of enriched GO terms was removed using the function simplify. The results are shown in Table 2 and Figures 4 and 5.

A total of 11 genes were enriched: two upregulated genes *HSPB1* and *DENR*, nine downregulated genes *RPS14*, *RPL35A*, *RPS24*, *RPL34*, *RPS15A*, *RPL27*, *RPL17*, *RPL31*, and *RSL24D1*. The main BP enriched by the 11 genes are as follows: translational initiation, SRP-dependent cotranslational protein targeting to membrane, cotranslational protein targeting to membrane, nuclear-transcribed mRNA catabolic process, nonsense-mediated decay, and establishment of protein localization to endoplasmic reticulum. This indicates that in addition to the degradation of abnormal transcripts, these genes are mainly involved in protein biosynthesis: protein translation, postsynthesis secretion, and targeted transport in the cytoplasm. The most important CC enriched by all the nine downregulated genes is cytosolic ribosome. Meanwhile, the nine downregulated genes are enriched the only one MF result: structural constituent of ribosome; in other words, they encode the ribosomal structural protein.

#### 3.1.4. KEGG Pathway Enrichment Analysis of Female-Specific DEGs

**Figure 6:**
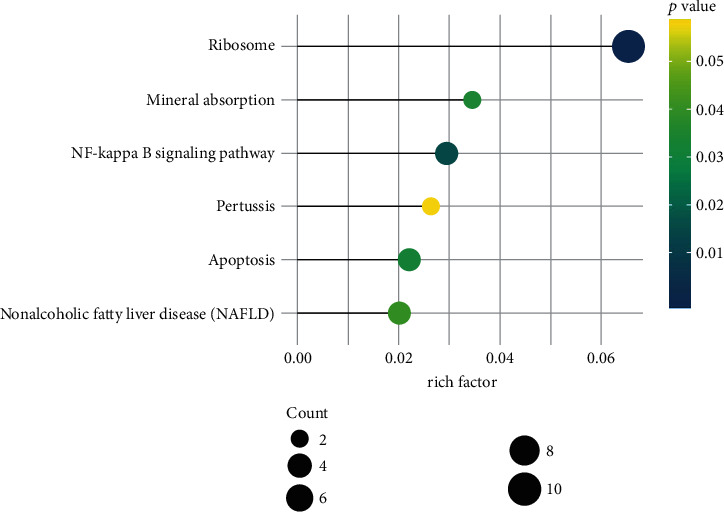
The top six enriched pathways with the highest rich factor of female-specific DEGs. Rich factor is equal to the gene count enriched into the pathway versus the total of genes contained in the pathway.

**Figure 7:**
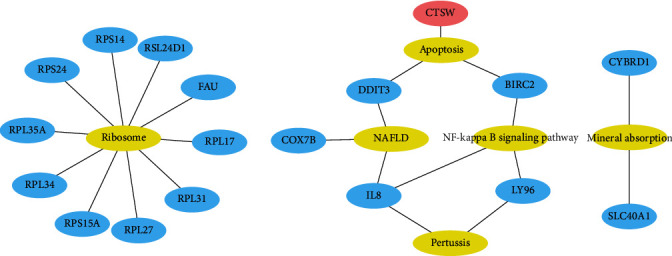
Significant pathway enrichment of female-specific DEGs. Yellow represents the signaling pathway, red represents the upregulated gene, and blue represents the downregulated gene. NAFLD: nonalcoholic fatty liver disease.

KEGG pathway enrichment analysis was performed using clusterProfiler in R package. A total of 18 genes are enriched in key signaling pathways, and all are downregulated genes except the only one *CTSW* that is upregulated. Six signaling pathways with higher rich factor than others are shown in Table 3 and Figure 6, which are ribosome, nuclear factor-­kappa B (NF-kappa B) signaling pathway, apoptosis, mineral absorption, nonalcoholic fatty liver disease, and pertussis. However, there was no significant difference in pertussis. The network diagram was drawn using the Cytoscape software and shown in Figure 7.

#### 3.1.5. Integration of the PPI Network and Functional Module Analysis

**Figure 8:**
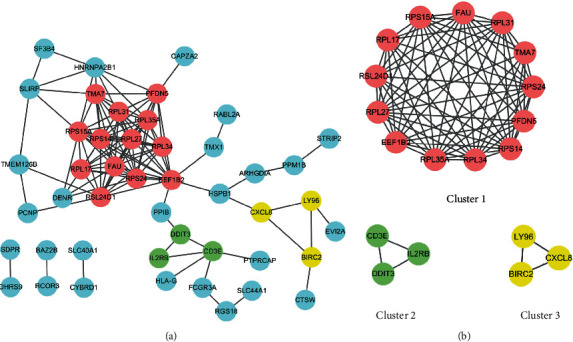
(a) PPI network of female-specific DEGs. (b) Three functional modules. Red represents the cluster 1 with score 12.167, 13 nodes, and 73 edges. Green represents the cluster 2 with score 3, 3 nodes, and 3 edges. Yellow represents the cluster 3 with score 3, 3 nodes, and 3 edges. *CXCL8* is also known as *IL8*.

The STRING online database was used to analyze the 79 female-specific DEGs, and the PPI network was visualized using the Cytoscape software, as shown in Figure 8(a). Using MCODE in Cytoscape to analyze the PPI network, three functional modules are clustered, as shown in Figure 8(b).

The cluster 1 included 13 genes, *FAU*, *RPL31*, *TMA7*, *RPS24*, *PFDN5*, *RPS14*, *RPL34*, *RPL35A*, *EEF1B2*, *RPL27*, *RSL24D1*, *RPL17*, and *RPS15A*, related to ribosome function, involving translation, elongation, and folding in the process of protein synthesis. The cluster 2 includes three genes, *CD3E*, *IL2RB*, and *DDIT3*, related to T cell adaptive immune and apoptosis. The cluster 3 includes three genes, *LY96*, *CXCL8* (also known as *IL8*), and *BIRC2*, related to NF kappa B signaling pathway.

#### 3.1.6. Selection of Key Genes

**Figure 9:**
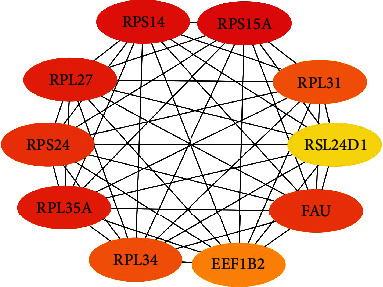
Top 10 key genes in the PPI network of the female-specific DEGs.

**Figure 10:**
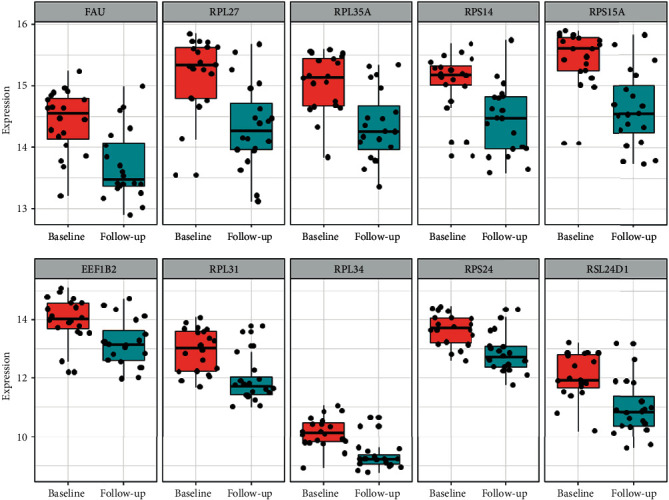
The expression of key genes in female IS patients based on follow-up vs. baseline.

The top key genes of the PPI network constructed were selected using CytoHubba in the Cytoscape software. The top key genes, which have higher MCC degree, including *RPS15A*, *RPS14*, *RPL27*, *RPL35A*, *FAU*, *RPS24*, *RPL31*, *RPL34*, *EEF1B2* and *RSL24D1*, are supposed to be more important than others because of their key position on the PPI network. Key genes are shown in Table 4 and Figure 9. The expression of key genes in female IS patients based on follow-up vs. baseline is shown in Figure 10.

### 3.2. Discussion

Sex influence IS epidemiology, pathophysiology, treatment efficacy, and outcome. A recent clinical study found that minocycline, which is an inhibitor of cell death, had significantly better neurological outcomes in IS male patients only, with no significant clinical improvement to female patients [14]. The study illustrated the importance of assessing sex as a variable in IS clinical research. Recent years, microarray analyses have been attempted in sex differences of human IS. Some previous studies have screened sexually dimorphic differences of gene expression by comparing male IS patients with females directly [15]. However, some DEGs were located on sex chromosomes that could represent transcript expression differences based on sex or differences based on disease being studied. To eliminate the confounding factors of sex chromosomes, the study of Tian et al. adopted a method that female IS patients were compared with female controls, and male IS patients were compared with male controls. The focus of the study of Tian et al. was the sexually dimorphic differences of gene expression of peripheral blood total RNA in untreated stroke patients within 3 hours after acute IS onset without discussion about the sexually dimorphic differences of IS progression [16]. The sex differences of gene expression of peripheral blood total RNA underlying in IS progression remain poorly understood [17, 18].

Our study is aimed at identifying the sex-specific DEGs in peripheral blood in IS progression, predicting potential biomarkers that can lead to sex-specific peripheral immune response and prognosis, and revealing potential sex-specific treatment targets. The GSE37587 datasets included 68 peripheral whole blood samples which were collected 20 IS women and 14 IS men within 24 hours (baseline) from known onset of symptom and again at 24-48 hours (follow-up) after onset. In order to have further information whether the gene expression of sex-specific DEGs and nonsex-specific was consistent, we screened two kinds of DEGs: nonsex specific and sex specific. Through the follow-up of 34 patients compared with the baseline of 34 patients, we got the nonsex-specific DEGs of the progression of IS. Adopted method of Tian et al., which follow-up of 20 females were compared with baseline of 20 females and follow-up of 14 males compared with baseline of 14 males, respectively, we got the sex-specific DEGs of the progression post-IS. Interestingly, the results show that there are 30 nonsex-specific DEGs, 79 female-specific DEGs, and none of male-specific DEGs. There are 23 overlaps between female-specific and nonsex-specific DEGs (Figure 1). Only 7 genes are unique to nonsex-specific DEGs. The only one gene upregulated in nonsex DEGs is also the one of 25 upregulated in female-specific DEGs (Table 1). It can be inferred that the female-specific DEGs are contributed to the most of the nonsex-specific DEGs. Therefore, we only need to analyze the female-specific DEGs to know the sexually dimorphic in the early recovery of IS patients. More than two-thirds of the female-specific DEGs are downregulated during the course of 24-48 hours after stroke (Figures 2 and 3).

GO analysis showed that the 11 genes enriched were mainly related to ribosomal and protein synthesis. Changes in the transcription level of ribosome-related genes have a broader impact on the downstream translation level. An in vitro experiment used ribosome profiling to explore the immediate transcription and translation change of neural cell gene expression to oxygen and glucose deprivation (OGD). It made a conclusion that the effect of OGD was widespread on translation than transcription [19]. It can be inferred that the expression of sex-specific ribosome differential genes in the early stage of stroke recovery may be the key underlying mechanism of sexually dimorphic difference in immune response. In addition, although the change of ribosome-related gene transcription level in peripheral blood reflects the peripheral immune response of granulocyte, lymphocytes, and monocytes to the greatest extent, there may also be genes coming from glial and nerve cells because of the damage of blood-brain barrier caused by stroke-induced inflammatory response. Therefore, the change of ribosome-related gene expression may also reflect the repair mechanisms of damaged brain.

The results of the KEGG enrichment analysis of female-specific DEGs are highly consistent with the GO analysis results. The most significant result is the ribosome followed by NF-kappa B signaling pathway and apoptosis and finally, the mineral absorption, nonalcoholic fatty liver disease, and pertussis. Ribosome is the place where biological protein synthesis takes place. It consists of two major components which the small ribosomal subunits in charge of reading the mRNA, and the large subunits in charge of joining amino acids to form a polypeptide chain. Each subunit consists of varieties of ribosomal proteins and one or more ribosomal RNA (rRNA) molecules. Ribosome-related genes downregulated lead to protein synthesis and secretion disorders. The NF-kappa B signaling pathway has a broad role in the regulation of numerous biological processes. NF-kappa B-dependent gene expression is required for proper development and formation of the immune system, innate immune responses, initiation of the adaptive immune response, pathogen recognition, inflammation, lymphoid organogenesis, transcription of cytokines, and immediate antimicrobial responses [20–22]. Downregulation of NF-kappa B signaling pathway leads to immunosuppression [23, 24]. Interestingly, we can see that the *DDIT3* encoding a proapoptotic transcription factor DNA damage-inducible transcript 3 (DDIT3, also known as CHOP) and the *BIRC2* encoding cIAP1, which is a member of the inhibitor of apoptosis family that inhibits apoptosis [25], are both enriched in the apoptosis pathway with downregulated. One possible explanation of these is a tug of war between proapoptosis and inhibit apoptosis under the ribosome downregulated. In addition, the only one upregulated *CTSW*, encoding a cysteine proteinase associated with cytotoxic T and natural killer cell [26, 27], is enriched in the apoptosis pathway. We can infer that these results are likely associated with sexually dimorphic immune response, repair, or other complex mechanisms in IS progression.

**Figure 11:**
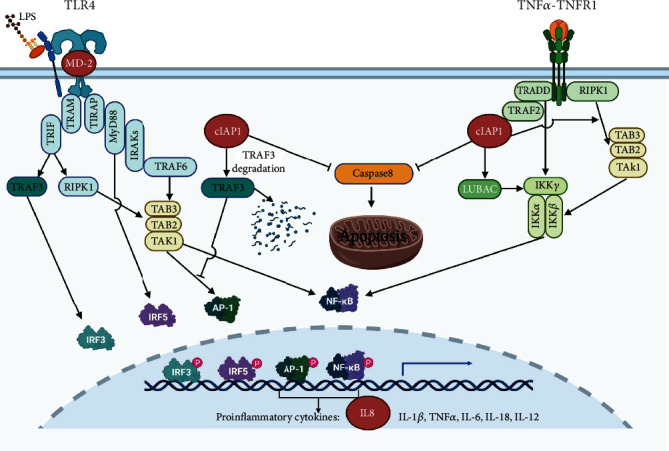
The mechanism of the cluster 3 DEGs in stroke-induced apoptosis and immunosuppression (created with http://BioRender.com/).

Three modules were obtained from the functional module analysis of the PPI network. The clusters 1 and 3 are basically consistent with the results of KEGG enrichment analysis. The cluster 1 is closely related to ribosome function, and the important genes involved will be discussed in the following selection of key genes. The cluster 2 includes three genes, *CD3E*, *IL2RB*, and *DDIT3*. CD3e molecule, epsilon (also known CE3E), encoded by *CD3E*, together with CD3-gamma, -delta, and -zeta and the T cell receptor alpha/beta and gamma/delta heterodimers, forms the T cell receptor-CD3 complex, which couples antigen recognition to several intracellular signal-transduction pathways. CD3E plays an essential role in T cell development and interleukin-2 production in T cells [28]. Interleukin-2 receptor subunit beta (also known as CD122), encoded by *IL2RB*, is involved in T cell-mediated immune responses. Activation of the receptor increases proliferation of CD8+ effector T cells [29]. The downregulation of *CD3E* and *IL2RB* suggests the downregulation of T cell adaptive immune response. *DDIT3* encodes DNA damage-inducible transcript 3 (DDIT3) that belongs to the family of CCAAT/enhancer-binding proteins (C/EBPs) [30]. It is also named C/EBP homologous protein (CHOP), which can form heterodimers with other C/EBP family proteins [31]. DDIT3 plays an important role in endoplasmic reticulum (ER) stress-induced apoptosis [30]. Under ER stress, DDIT3 is a transcriptional repressor to downregulate the expression of antiapoptotic genes such as *BCL-2* and a transcriptional activator to upregulate expression of proapoptotic genes such as *BAX* [30, 32]. Therefore, cluster 2 is related to T cell adaptive immune and apoptosis. The cluster 3 includes three genes, *LY96*, *CXCL8* (also known as *IL8*), and *BIRC2*. The MD-2 protein (also known as lymphocyte antigen 96), encoded by *LY96*, associates with toll-like receptor 4 (TLR-4) on the cell surface and confers responsiveness to lipopolysaccharide (LPS) [33]. LPS is the ligand of TLR-4, and via p38, JNK, and NF-kappa B signaling pathways, its interaction results in activation of proinflammatory cytokines such as TNF*α*, IL-1*β*, IL-6, IL-18, and IL-12 [34] and chemokines such as IL8. IL8 (also known as CXCL8), encoded by *CXCL8* (*IL8*), is a chemokine produced by macrophages mainly. Its primary function is to induce chemotaxis in target cells, primarily neutrophils, causing them to migrate toward the site of infection. cIAP1, encoded by *BIRC2*, is a member of the inhibitor of apoptosis family that inhibits apoptosis by interfering with the activation of caspases [35]. Meanwhile, cIAP1 also can set a balance between NF-kappa B transcription factor and apoptosis signaling downstream of tumor necrosis factor (TNF) receptor superfamily members by acting as ubiquitin E3 ligases for substrates that are part of the TNF receptor complex [36]. In addition, on the signalling route of TLR4­ mediated production of proinflammatory cytokines, cIAPs are also required for ligand­ induced degradation of TNF receptor-associated factor 3 (TRAF3), which functions as an inhibitor of mitogen-activated protein kinase (MAPK) activation and inflammatory cytokine production [37]. It can be concluded that the downregulation of the cluster 3 reveals the downregulation of NF-kappa B signaling pathway, the reduction of cytokine, and decreased inhibition of apoptosis (Figure 11).

The results of key gene analysis further confirm the results of GO, KEGG, and functional module analysis. 10 top key genes screened, which all of them are downregulated, related to ribosome or translation. We obtained the basic information of these genes through the Human Gene Database GeneCards (https://www.genecards.org/). *RPS14*, *RPS15A*, and *RPS24* encode 40S ribosomal proteins. *FAU* encodes a fusion protein consisting of the ubiquitin-like protein fubi at the N terminus and ribosomal protein S30 at the C terminus, which is posttranslationally processed to generate free fubi and free ribosomal protein S30. Whereas the function of fubi is currently unknown, ribosomal protein S30 is a component of the 40S subunit of the cytoplasmic ribosome and displays antimicrobial activity [38]. *RPL27*, *RPL31*, *RPL34*, and *RPL35A* encode 60S ribosomal proteins. The above key genes participate in the structure of ribosome and protein synthesis. *RSL24D1* encodes probable ribosome biogenesis protein (RLP24), which localizes to the nucleolus and is thought to play a role in the biogenesis of the 60S ribosomal subunit. *EEF1B2* encodes a translation elongation factor, named eukaryotic translation elongation factor 1 beta 2, which involved in the transfer of aminoacylated tRNAs to the ribosome. We infer that the downregulation of key genes will affect the protein synthesis of peripheral blood immune cells and thus cause immunosuppression.

As early as 1974, immunosuppression was found to be the most common complication of acute stroke [39]. Subsequently, a large number of studies showed that the main characteristics of SIDS are rapid and lasting immunosuppression: the decrease of monocyte activity, the decrease of T-lymphocyte count, the imbalance of Th1/Th2 [40], and the significant apoptosis of immune cells in peripheral immune organs (such as spleen and lymph nodes) [41, 42]. However, there are few reports about sexually dimorphic differences in SIDS. In this study, female IS patients show significant immunosuppressive response in the early recovery: ribosomal and NF-kappa B signaling pathway downregulation, T cell immune activity downregulation, and the level downregulation between proapoptosis and inhibit apoptosis, especially ribosome downregulation. However, the control male IS patients show few significant changes in peripheral gene expression. These may have an explanation why there are sex differences in stroke outcomes. At the same time, we observed the age of 20 female IS patients with mean age of 72.15 ± 15.951 years, which the youngest is 43 years and the oldest is 92 years. Most of them belong to the elderly women. Therefore, the occurrence of such differences may be more related to biological differences [43], rather than the differences caused by estrogen.

After a comprehensive exploring of female-specific DEGs, we studied main nonsex-specific DEGs. According to the introduction of GeneCards, *RPL39* and *RPL26* encode 60S ribosomal proteins which work at the structure of ribosome and protein synthesis. The downregulation of these genes may affect the protein synthesis of peripheral blood immune cells. *AKAP7* encodes a member of the A-kinase anchoring protein (AKAP) family, which are molecular scaffolding proteins binding to a regulatory subunit (RII) of cAMP-dependent protein kinase A (PKA), directing the kinase to discrete subcellular locations [44, 45]. Alternatively spliced transcript variants from the *AKAP7* (AKAP15/18) are essential components of neuronal and cardiac phosphatase complexes, ion channels, cardiac Ca^2+^ handling, and renal water transport [46]. A new study found that early heightened expression levels of *AKAP7* were significantly associated with poststroke blood-brain barrier (BBB) disruption 24 hours posthospital admission, which suggested that *AKAP7* may be a prognostic biomarker for poststroke BBB complications [47]. In this study, the downregulation of AKAP7 may indicate that the severity of BBB damage at post-IS 48 hours is lower than at 24 hours; in other word, it is within post-IS 48 hours that the BBB damage begins to repair. The role of other nonsex-specific DEGs on the development and prognosis of IS remains to be further studied.

The innovation of this study is to take the lead in analyzing the difference of gene expression between men and women in the early stage of stroke recovery through high-throughput biochip transcriptome datasets and to find that women are more responsive in stroke-induced immunosuppression, and ribosome-related genes play the most important roles in stroke-induced immunosuppression. This finding provides a new insight to evaluate the prognosis and potential accurate treatment targets. Ribosome-related genes may be a novel prognostic biomarker and a potential therapeutic target for IS. However, the specific mechanism of ribosome in SIDS needs to be further studied in vitro and in vivo.

## 4. Conclusions

Sex difference of ribosome in stroke-induced peripheral immunosuppression may be the potential mechanism of sex disparities in outcome after IS, and women are more likely to have SIDS after IS. *RPS14*, *RPS15A*, *RPS24*, *FAU*, *RPL27*, *RPL31*, *RPL34*, *RPL35A*, *RSL24D1*, and *EEF1B2* may be novel prognostic biomarkers and potential therapeutic targets for IS.

## Figures and Tables

**Table 1:** Nonsex- and sex-specific DEGs.

DEGs	Regulated	Gene symbol
Nonsex-specific	Upregulated	*CDC14B*
	Downregulated	*LY96*, *SDPR*, *IL8, COMMD6*, *C15orf15*, *RPL31*, *CCDC72*, *COX7B*, *EVI2A*, *RPL17*, *AKAP7*, *RGS18*, *EEF1B2*, *RPL27*, *TXNDC*, *RPS24*, *RPS15A*, *HES4*, *CAPZA2*, *RPL34*, *BAZ2B*, *BIRC2*, *C14orf156*, *HINT1*, *ARL5A*, *MBNL2*, *MASK*, *RPL39*, *RPL26*
Female-specific	Upregulated	*CDC14B*, *FCGR3A*, *HLA-G*, *AMY1A*, *GIMAP7*, *PTPLAD2*, *USP49*, *HNRPA2B1*, *ZNF557*, *RABL2A*, *LDHB*, *ARHGDIA*, *CTSW*, *PTPRCAP*, *FAM40B*, *CD3E*, *PPIB*, *LOC441743*, *RPL23AP13*, *IL2RB*, *MBD4*, *HSPB1*, *SF3B4*, *LOC400986*, *DENR*
	Downregulated	*LY96*, *SDPR*, *IL8*, *COMMD6*, *C15orf15*, *CCDC72*, *RGS18*, *RPL31*, *COX7B*, *CAPZA2*, *C21orf7*, *RPL17*, *TXNDC*, *EVI2A*, *RPL27*, *BAZ2B*, *RPS15A*, *EEF1B2*, *RPL34*, *RPS24*, *PFDN5*, *C14orf156*, *BIRC2*, *TMEM55A*, *HIST1H4H*, *GPR160*, *FAU*, *HSH2D*, *MMD*, *ARL5A*, *OXR1*, *RPL35A*, *PCNP*, *KIAA0143*, *LSM1*, *DHRS9*, *SLC44A1*, *PPM1B*, *SNRK*, *ANKRD49*, *CKS2*, *RPS14*, *RCOR3*, *TMEM126B*, *CYBRD1*, *CLEC4D*, *CCPG1*, *MGC4677*, *WDFY1*, *AP1S2*, *DDIT3*, *ST8SIA4*, *SLC40A1*, *SNX16*
Male-specific	Upregulated	None
	Downregulated	None

Abbreviation: DEGs: differentially expressed genes. Note: *C15orf15* is also known as *RSL24D1*.

**Table 2:** Enriched GO terms of female-specific DEGs.

Category	ID	Term	Gene ratio	*p* value	*p* adjust	*q* value	Gene
BP	0006413	Translational initiation	10/73	3.90*E*-09	3.80*E*-06	3.63*E*-06	*RPL34*, *RPS15A*, *HSPB1*, *RPS24*, *RPL27*, *RPL31*, *RPL35A*, *RPL17*, *RPS14*, *DENR*
BP	0006614	SRP-dependent cotranslational protein targeting to membrane	8/73	7.59*E*-09	3.80*E*-06	3.63*E*-06	*RPL34*, *RPS15A*, *RPS24*, *RPL27*, *RPL31*, *RPL35A*, *RPL17*, *RPS14*
BP	0006613	Cotranslational protein targeting to membrane	8/73	1.02*E*-08	3.80*E*-06	3.63*E*-06	*RPL34*, *RPS15A*, *RPS24*, *RPL27*, *RPL31*, *RPL35A*, *RPL17*, *RPS14*
BP	0000184	Nuclear-transcribed mRNA catabolic process, nonsense-mediated decay	8/73	2.18*E*-08	4.62*E*-06	4.41*E*-06	*RPL34*, *RPS15A*, *RPS24*, *RPL27*, *RPL31*, *RPL35A*, *RPL17*, *RPS14*
BP	0072599	Establishment of protein localization to endoplasmic reticulum	8/73	2.49*E*-08	4.62*E*-06	4.41*E*-06	*RPL34*, *RPS15A*, *RPS24*, *RPL27*, *RPL31*, *RPL35A*, *RPL17*, *RPS14*
BP	0019080	Viral gene expression	9/73	5.61*E*-08	8.95*E*-06	8.54*E*-06	*RPL34*, *RPS15A*, *RPS24*, *RPL27*, *RPL31*, *RPL35A*, *RPL17*, *RPS14*, *DENR*
BP	0019083	Viral transcription	8/73	4.43*E*-07	4.49*E*-05	4.29*E*-05	*RPL34*, *RPS15A*, *RPS24*, *RPL27*, *RPL31*, *RPL35A*, *RPL17*, *RPS14*
CC	0022626	Cytosolic ribosome	9/74	3.61*E*-10	6.28*E*-08	5.81*E*-08	*RPL34*, *RPS15A*, *RPS24*, *RPL27*, *RPL31*, *RPL35A*, *RSL24D1*, *RPL17*, *RPS14*
CC	0044445	Cytosolic part	9/74	3.57*E*-07	1.55*E*-05	1.44*E*-05	*RPL34*, *RPS15A*, *RPS24*, *RPL27*, *RPL31*, *RPL35A*, *RSL24D1*, *RPL17*, *RPS14*
MF	0003735	Structural constituent of ribosome	9/73	1.42*E*-07	2.54*E*-05	2.45*E*-05	*RPL34*, *RPS15A*, *RPS24*, *RPL27*, *RPL31*, *RPL35A*, *RSL24D1*, *RPL17*, *RPS14*

Abbreviation: BP: biological process; CC: cellular component; MF: molecular function.

**Table 3:** KEGG pathway enrichment analysis of female-specific DEGs.

ID	Description	Gene ratio	BgRatio	*p* value	*p* adjust	Genes
hsa03010	Ribosome	10/41	153/8016	3.23*E*-09	3.48*E*-07	*RPL34*, *RPS15A*, *RPS24*, *RPL27*, *FAU*, *RPL31*, *RPL35A*, *RSL24D1*, *RPL17*, *RPS14*
hsa04064	NF-kappa B signaling pathway	3/41	102/8016	0.015027	0.54667	*LY96*, *BIRC2*, *IL8*
hsa04210	Apoptosis	3/41	136/8016	0.031862	0.54667	*DDIT3*, *BIRC2*, *CTSW*
hsa04978	Mineral absorption	2/41	58/8016	0.035231	0.54667	*SLC40A1*, *CYBRD1*
hsa04932	Nonalcoholic fatty liver disease (NAFLD)	3/41	149/8016	0.040123	0.54667	*DDIT3*, *COX7B*, *IL8*
hsa05133	Pertussis	2/41	76/8016	0.057363	0.54667	*LY96*, *IL8*

**Table 4:** Top 10 key genes in the PPI network of the female-specific DEGs ranked by MCC method.

Rank	Gene symbol	Gene description	MCC score	Log_2_ FC
1	*RPS15A*	Ribosomal protein S15a	4354708	-0.83625
2	*RPS14*	Ribosomal protein S14	4354704	-0.63886
3	*RPL27*	Ribosomal protein L27	4354680	-0.84896
3	*RPL35A*	Ribosomal protein L35a	4354680	-0.68986
5	*FAU*	FAU ubiquitin like and ribosomal protein S30 fusion	4354584	-0.72147
5	*RPS24*	Ribosomal protein S24	4354584	-0.81476
7	*RPL31*	Ribosomal protein L31	4354560	-1.0045
7	*RPL34*	Ribosomal protein L34	4354560	-0.82663
9	*EEF1B2*	Eukaryotic translation elongation factor 1 beta 2	3991683	-0.82928
10	*RSL24D1*	Ribosomal L24 domain containing 1	3628802	-1.0838

## Data Availability

The data used to support the findings of this study are included within the article.
